# Modified Open Suprapectoral EndoButton Tension Slide Tenodesis Technique of Long Head of Biceps with Restored Tendon Tension–Length

**DOI:** 10.2174/1874325001711010281

**Published:** 2017-03-31

**Authors:** Jagadish Prabhu, Mohammed Khalid Faqi, Rashad Khamis Awad, Fahad Alkhalifa

**Affiliations:** Bahrain Defence Force Hospital - Royal Medical Services, Riffa, Kingdom of Bahrain

**Keywords:** Biceps rupture, Eendobutton, Ttendon sliding techniques/instrumentation, Ttenodesis

## Abstract

**Background:**

The vast majority of biceps tendon ruptures occurs at the proximal insertion and almost always involves the long head. There are several options for long head of biceps (LHB) tenodesis with advantage and disadvantages of each technique. We believe that the suprapectoral LHB tenodesis described in this article enables the restoration of the anatomic length-tension relation in a technically reproducible manner, when following the guidelines set forth in this article, and restores biceps contour and function adequately with a low risk of complications.

**Method:**

We present a case of a young man who had a sudden jerk of his flexed right elbow, while involved in water skiing sports and sustained complete rupture of proximal end of long head of biceps tendon. In this article, we describe a modified surgical technique of open supra-pectoral long head of biceps tenodesis using an EndoButton tension slide technique, reproducing an anatomic length-tension relationship.

**Results:**

By the end of one year, patient regained symmetrical muscle bulk, shape and contour of biceps compared to other side. There were no signs of dislodgement or loosening of the EndoButton on follow-up radiographs. He regained full muscle power in the biceps without any possible complications, such as humeral fracture, infection, or nerve injury, associated with this technique.

**Conclusion:**

This technique is a safe, easy to reproduce, cost-effective, less time consuming and an effective method that uses a small drill hole, conserving bone, minimizing trauma to the tendon, and decreasing postoperative complications. It does not need any special instrumentation and is suitable especially for use in centers where arthroscopy facility or training is not available.

## INTRODUCTION

Rupture of the long head of the biceps (LHB) is usually seen in older adults, often in conjunction with rotator cuff tears, superior labrum anterior to posterior (SLAP) lesions, or tendinosis secondary to chronic subacromial impingement [1, 2]. In rare instances, proximal biceps ruptures are seen as a result of high-energy trauma and may result from an overloading flexion force or flexion against the forced extension [3].

There are several options for long head of biceps tenodesis with advantages and disadvantages of each technique. We report a surgical technique of supra-pectoral long head of biceps tenodesis using a cortical button reproducing an anatomic length-tension relationship.

## CASE PRESENTATION

**Fig. (1):**
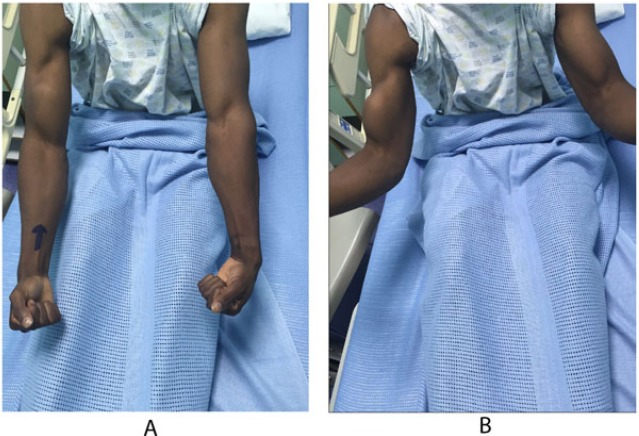
Clinical picture showing - rupture of the LHB, denoted by a typical 'popeye sign' with a dropped biceps muscle.

A 28 years old healthy man had a sudden jerk of his flexed right elbow to extension by the tension of the towrope as the boat accelerated away while involved in water skiing sports. He presented to the emergency department with severe pain in the anterior compartment of his right arm and a “Popeye” deformity, with clear rupture of the long head of the biceps (Figs. **1A**, **1B**). The plain radiographs of the left arm, elbow, and forearm did not reveal any fractures or dislocations. Magnetic resonance imaging showed complete proximal rupture of long head of biceps tendon.

## SURGICAL TECHNIQUE

The patient is placed in the beach-chair position with adequate clearance of the shoulder and forearm fixed to a limb positioner (Tri-Mano), which can be used for controlling and positioning the arm. The entire arm is sterilely prepared so that it can be freely manipulated during surgery. The arm is suspended in approximately 30° of abduction, 15° of forward flexion, and neutral rotation.

**Fig. (2):**
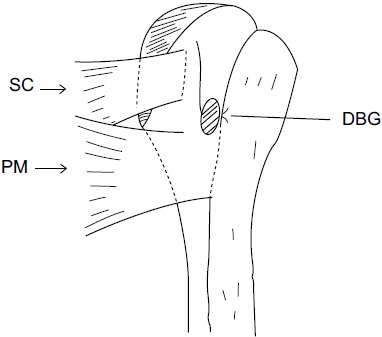
Line drawing showing the preferred location for tenodesis of the long head of the biceps (LHB) tendon at the distal bicipital groove (DBG) with an open supra-pectoral technique. SC: Subscapularis; PM: Pectoralis Major.

Using a proximal delto-pectoral skin incision, the distal part of the bicipital groove (DBG) is exposed, retracting the deltoid-pectoral complex superolaterally with a Hohmann retractor, while the short head of the biceps brachii and the coracobrachialis muscles are retracted medially with a right angled retractor. Adequate visualization of the distal part of the biceps groove is paramount. One should avoid vigorous medial retraction to protect the musculocutaneous nerve. The site for drilling is identified at the distal-most aspect of the biceps groove (Fig. **2**).

**Fig. (3):**
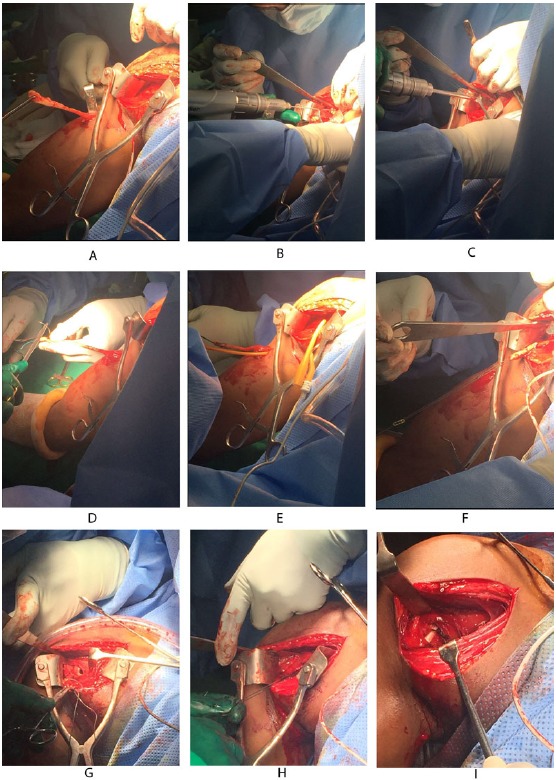
Intraoperative photographs of the Tension-slide technique of proximal biceps tendodesis using an EndoButton reproducing anatomical length-tension relationship. A, The proximal end of the biceps tendon is retrieved through second subpectoral incision; B, Guide wire passed through the distal bicipital groove (DBG); C, making a drill hole with cannulated reamer over the guide wire; D, proximal end of biceps tendon sutured by Krachow suturing technique using No.5 synthetic polyester suture (Ethibond); E, getting sutured biceps tendon through delto-pectoral incision using smallest size foley’s catheter; F, passing suture limbs through 4 holed EndoButton; G,The EndoButton is loaded onto the Button deployment device and passed through bone tunnel; H, Pulling on the sutures shuttles the LHB tendon into the humerus; I, end of the procedure showing proximal biceps tenodesis at distal bicipital groove.

If the proximal end of biceps tendon could not be traced with this approach, then we need to make another 3cm incision in the subpectoral region, centered over the inferior border of the pectoralis major tendon. Superficial soft tissue is dissected to expose the fascia over the inferior border of the pectoralis major muscle. The fascia is incised from the lower border of the pectoralis muscle distally along the coracobrachialis and biceps muscles. The longitudinal, white tendon of the biceps tendon is identified and pulled out of the incision (Fig. **3A**).

**Fig. (4):**
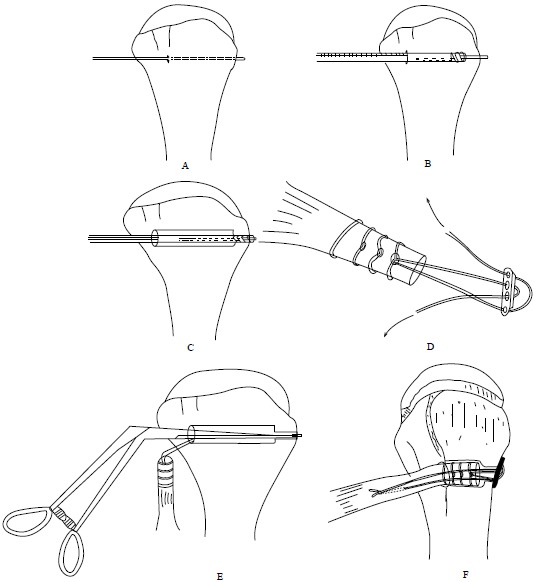
Illustrations of the surgical steps in the Tension-slide technique of proximal biceps tendodesis using an EndoButton reproducing anatomical length-tension relationship A, Passing the guide wire through distal bicipital groove (DBG) B, Reaming the anterior cortex and the intramedullary canal over the guide wire using a calibrated cannulated drill. C, Drilling the posterior cortex over the guide wire using an EndoButton drill. D, Passing suture thread limbs through the 4-holed EndoButton. E, Inserting a threaded EndoButton through the bone tunnel. F, Tightening the suture threads and flushing the EndoButton on the posterior cortex and tying the knot under tension after passing the suture ends through the proximal end of the long head biceps tendon, once it is shuttled through the bone tunnel to reinforce the fixation.

At this stage, we go back to our first incision site to prepare the tenodesis site. The distal part of the bicipital groove (DBG) is identified and cleared off soft tissues. A 2.7mmx 15” guide wire is drilled at an angle of 45º through the center of DBG. Care should be taken to avoid skydiving off the edge of the humeral shaft (Fig. **3B**). The guide wire should be stopped as soon as the pin penetrates the posterior cortex (Fig. **4A**). This is followed by drilling the anterior cortex over the guide wire with a calibrated cannulated drill, the size of which is the same as the measured diameter of the tendon (in our case, a 7-mm drill for a 7-mm tendon) (Figs. **3C**, **4B**). Care is taken to only drill up to the posterior cortex without engaging the cortex or plunging through. Depth of this bone tunnel is measured with the aid of a calibrated drill (TD- Tunnel Depth). Usually it is around a 20-25mm tunnel in adults. The hole should be lined up at the base of the biceps groove and cleared of soft tissue. Finally, the posterior cortex is drilled through with a 4.5-mm drill to allow for the passage of the cortical button (Fig. **4C**).

Next step is the preparation of the proximal biceps tendon for tenodesis. We believe that restoring the native length-tension relation of the LHB is an important and often difficult step when performing tenodesis of this structure. With under-tensioning of the LHB, the procedure may result in a persistent biceps deformity, early muscle fatigue, and subjective cramping [4, 5]. With over-tensioning of the LHB, the pullout forces at the site of tenodesis increase, potentially leading to fixation failure.

**Fig. (5):**
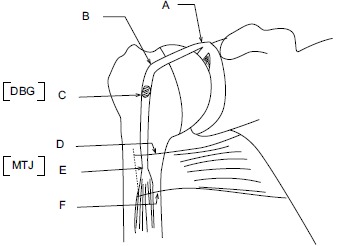
Schematic drawing showing location of landmarks in relation to the long head of the biceps. (A) Labral origin; (B) Superior bicipital groove; (C) Distal bicipital groove (DBG); (D) Superior border of pectoralis tendon; (E) Musculotendinous junction (14); and (F) Inferior border of the pectoralis tendon.

**Fig. (6):**
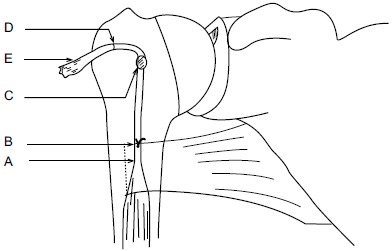
Illustrations showing method of calculating the required tendon length to reproducing anatomical length-tension relationship. (A) Marking musculotendinous junction; (B) 22mm proximal to mark A; a simple stay suture knot applied at this location with superior border of pectoralis tendon; (C) mark on biceps tendon at DBG level; (D) mark proximal to mark C, at a distance equal to tunnel depth (TD) i.e if TD is 25mm, then mark D is 25mm proximal to mark C; (E) Proximal end of biceps tendon. D-E: is the segment of tendon to be resected. Tendon suturing should be started from mark D and then two suture limbs from tendon passed through the 4 holed EndoButton, as explained above.

**Fig. (7):**
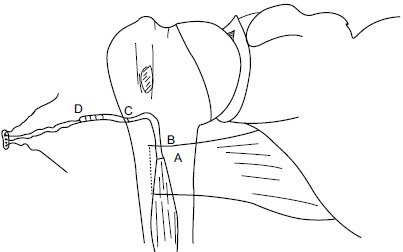
Illustrations showing method of calculating the required tendon length to reproducing anatomical length-tension relationship. (A) Marking musculotendinous junction; (B) 22mm proximal to mark A; a simple stay suture knot applied at this location with superior border of pectoralis tendon; (C) mark on biceps tendon at DBG level; (D) mark proximal to mark C, at a distance equal to tunnel depth (TD) i.e if TD is 25mm, then mark D is 25mm proximal to mark C; (E) Proximal end of biceps tendon. D-E: is the segment of tendon to be resected. Tendon suturing should be started from mark D and then two suture limbs from tendon passed through the 4 holed EndoButton, as explained above.

Hereby, we describe a novel technique to estimate the required tendon length and calculate the tendon resection length (Figs. **5**, **6**, **7**). Jarrett *et al.* [6], found that the musculo-tendinous junction (MTJ) of the biceps lies 22 mm distal to the upper border of the pectoralis major tendon and 31 mm proximal to the lower border of the pectoralis major tendon. The MTJ is identified and marked (A) (Fig. **6**). Another point (marked B) Fig. (**6**) lies 22mm above the MTJ. The tension on the tendon is released, ensuring that mark B is at the upper border of the pectoralis major tendon. At this point, a stay suture knot is applied through the biceps tendon and the upper border of the pectoralis major tendon (mark B).

Now, the tendon end to the DBG is retrieved using the smallest size Foleys catheter. Keep the tendon over the tunnel, without any kinking, and mark (C) on the tendon at DBG (Fig. **6**). Make another mark (D) on the tendon at a distance equal to measured tunnel depth (TD) (Fig. **6**). Hence, the required tendon length for this procedure is A-D (Fig. **6**). The tendon is resected at mark D. Now, the tendon end is pulled back through distal incision for suturing. This is because it is easy to suture the tendon when we have enough length of tendon to hold and know the rotational alignment of the tendon.

A number-5 synthetic polyester suture (Ethibond Excel, Ethicon Inc; Johnson and Johnson, USA) is woven into the proximal biceps tendon using the Krackow technique or whipstitch suture technique (Fig. **3D**). The tendon end to the DBG is retrieved again using the smallest size Foleys catheter (Fig. **3E**). We used a 4-holed Endo Button without continuous loop (4.0 mm × 12 mm Endo-Button CL Ultra, Smith and Nephew, Andover, MA, USA). One limb of suture from the tendon end was threaded through the central two holes of the Endo Button (inside out – outside in). The other suture end from the tendon was passed through the terminal two holes of the Endo Button in a reverse manner i.e. from outside in – inside out (Figs. **4D**, **7**, **3F**). It is important to make certain that the suture limbs are not tangled.

The Endo Button is loaded onto the Button deployment device and passed through bone tunnel (Figs. **4E**, **3G**). Once the button clears the posterior cortex, the deployment instrument can be removed while the button deploys itself, locking into place on the posterior cortex. Pulling on the sutures shuttles the LHB tendon into the humerus. The sutures are tensioned until the tendon is in contact with the posterior cortex (Fig. **3H**). Finally, we advocate using a free needle to pass one suture through the tendon and tie down to the second suture to reinforce the fixation (Figs. **4F**, **3I**).

**Fig. (8):**
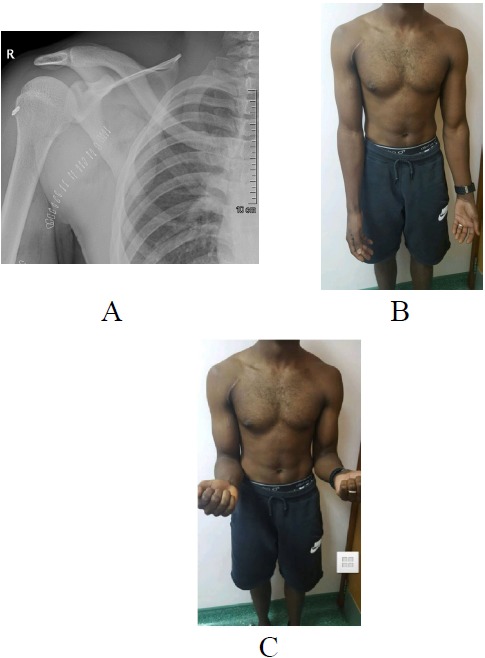
(A) Post-operative anterolateral radiograph showing the ideal position of the EndoButton at the posterior cortex of the proximal humerus, in line with the DBG. Clinical images (B) and (C) taken 1-year post biceps tenodesis showing a well healed surgical scar as well as symmetrical muscle bulk.

Unlike the use of the Biceps Button in the elbow, an additional interference screw is not required to achieve adequate fixation of the tendon. Rather, an additional suture is passed through the tendon of the LHB once it is shuttled through the humerus. This provides additional fixation strength to withstand physiologic loads. At this stage, cut the stay suture applied at superior border of pectoralis major tendon to complete the procedure. Intra-operative radiograph are taken to confirm the position of Endo Button. Wound wash is given and closed in layers. Cuff and collar is applied to support the arm for the first two weeks. Postoperative radiographs were obtained to assess the tenodesis location (Fig. **8A**).

## POST-OP REHABILITATION AND FOLLOW-UP

From post- operative day one, we started with pendulum, range of motion exercises for shoulder joint and passive flexion – active extension exercises for elbow joint. Active elbow flexion without resistance started from 3^rd^ week onwards. Active elbow flexion with gradual increase in resistance as pain tolerated started from 6 weeks. Patient was followed-up at 2 weeks, 1-3-6 months and 1 year post-operatively.

## RESULT

By the end of one year, patient regained symmetrical muscle bulk, shape and contour of biceps compared to other side (Figs. **8B**, **8C**). There was no signs of dislodgement or loosening of the Endo Button on follow-up radiographs. He regained full muscle power in the biceps without any possible complications, such as humeral fracture, infection, or nerve injury, associated with this technique.

## DISCUSSION

In this article we described an open technique for supra-pectoral biceps tenodesis using a bi-cortical Endo Button as a fixation tool, whilst maintaining length-tension relationship for best patient outcome with least possible complications.

Proximal biceps tenodesis has been previously described using different implants and surgical techniques [7-12]. Most of these articles described biceps tenodesis techniques for post surgical tenotomy for tendinitis/tendinosis or degenerative rupture/tearing of the biceps tendon, subluxation or associated with SLAP lesions. In such cases, it is ideal to go for an all-arthroscopic biceps tenodesis procedure. However, in case of traumatic complete rupture of the long head of biceps tendon with distal retraction into the mid arm, there are two ideal sites for fixation; either sub-pectoral or supra-pectoral tenodesis. In such cases, an open, double window approach for exploration, retrieval and fixation/tenodesis is done.

The disadvantages of the arthroscopic technique are the need for special instrumentation, use of hardware, costs, and inherent technical difficulties [13]. The open technique is useful in situations where arthroscopy facilities are unavailable or where surgeon is unfamiliar with the arthroscopic technique, which needs a long learning curve.

The open technique enables better visualization of the entire biceps tendon and its sheath, and does not require arthroscopic instruments. It enables examination of the biceps sheath and distal biceps tendon for unidentified tears, synovitis, and fibrosis. However, its use is limited due to its low primary stability, the need for a deltopectoral incision, postoperative pain, and cosmesis [7]. In our case, we found that the proximal end of LHB tendon was retracted to infra-pectoral region using an MRI. This mandates an open access to retrieve the tendon back to the preferred site of tenodesis.

We opted for a supra-pectoral tenodesis instead of a sub-pectoral tenodesis for the following reasons. Firstly, it is close to the origin of the biceps tendon (at superior labrum), thus the length of tendon resection is less, compared to a subpectoral tenodesis [14]. Since considerable length of tendon is preserved, even if this tenodesis fails in future, a subpectoral tenodesis could still be done as a second option. Secondly, there has been recent literature published on the neurovascular structures at risk during the subpectoral tenodesis [8, 13, 15-17]. The most common structure at risk is the radial nerve from drilling the humerus posteriorly. Thirdly, a cadaveric biomechanical analysis by Patzer *et al.* [18], investigated different methods and sites of fixation for LHB tenodesis. Fixation within the suprapectoral region was compared with the more distal, subpectoral location. The authors found that the highest ultimate load to failure was shown with fixation within the suprapectoral region. In another biomechanical study, there was no significant difference in peak failure load, displacement at peak load, or displacement after cyclic testing between the suprapectoral technique and the subpectoral tenodesis technique [19]. Fourthly, recent anatomic studies by Jarrett *et al.* [6], has questioned the ability of the subpectoral tenodesis to adequately restore the LHB length-tendon relation. They found that the musculotendinous junction of the native LHB is far closer to the superior border of the pectoralis major tendon than its inferior border, making restoration of the anatomic LHB tension through a subpectoral approach a near technical impossibility. Moreover, sub-pectoral tenodesis has been associated with a risk of stress fracture of the proximal humerus [20]. Jarrett *et al.* [6], found that, on average, the musculocutaneous nerve was 3.47 cm medial to the LHB at the superior border of the pectoralis tendon and 2.6 cm medial to the LHB at the musculocutaneous junction. These data show that the musculocutaneous nerve is further away from the LHB when performing our described procedure as compared with an open subpectoral tenodesis. Fifthly, David M *et al.* [21], suggested a distal bicipital groove tenodesis location may decrease the incidence of persistent postoperative pain at the bicipital groove.

Many techniques have been described in literature for proximal biceps tenodesis including interference screws, suture anchors, soft-tissue fixation and bone tunnel, amongst others [7-12]. A study conducted by Kusma M *et al.,* comparing 5 different proximal biceps tenodesis techniques (using suture anchor, bone tunnel, keyhole, interferential screw and ligament washer), the interferential screw fixation was superior in terms of ultimate load to failure and gap formation [22]. Nonetheless, another study reported increased gap formation after interferential screw fixation [23]. The tendon can fail at the bone-tendon interface or because of the pullout of the interferential screw or anchors, or suture breakage at the eyelet [24]. At the site of tenodesis (supra-pectoral), a weak bone can be problematic with interferential screw fixation [13].

Other techniques, such as interference screws, use compression to achieve fixation. Conversely, the cortical button technique uses a “sling and tensioning” that may be less traumatic to the tendon fibers at the time of fixation, thus decreasing the risk of failure. Though rare, there are reports of interference screws failing at the tendon-screw interface [25].

To the best of our knowledge, there is no clinical or cadaveric study comparing the endobutton fixation with other techniques for proximal biceps tenodesis. On the other hand, in distal biceps tenodesis, the endobutton technique proved to be better than other techniques [26-28].

## CONCLUSION

This technique is a safe, easy to reproduce, cost-effective, less time consuming and an effective method that uses a small drill hole, conserving bone, minimizing trauma to the tendon, and decreasing postoperative complications. It does not need any special instrumentation and is suitable especially for use in centers where arthroscopy facility or training is not available.

We believe that the suprapectoral LHB tenodesis, described in this article, enables the restoration of the anatomic length-tension relation in a technically reproducible manner when following the guidelines set forth in this article. It restores biceps contour and function adequately with a low risk of complications. However, long-term clinical outcomes studies are still needed.

Our surgical technique and recommendations will help other orthopaedic surgeons when preparing for open supra-pectoral biceps tenodesis procedures.

## LIST OF ABBREVIATIONS

DBG: = Distal part of the bicipital groove
LHB: = Long head of biceps
MTJ: = Musculo-tendinous junction
SLAP: = Superior labrum anterior to posterior
TD: = Tunnel Depth
